# Study of Association Between Different Coronary Artery Disease Presentations and Its Effect on Short-Term Mortality, Readmission, and Cost in Patients Undergoing Percutaneous Coronary Interventions

**DOI:** 10.7759/cureus.16862

**Published:** 2021-08-03

**Authors:** Mansoor Ahmad, Muhammad Asghar, Udit Joshi, Nathan A Neilson, Michael Tye, Chirag Divecha, Minchul Kim, Sudhir Mungee

**Affiliations:** 1 Cardiology, University of Illinois College of Medicine at Peoria, Peoria, USA; 2 Internal Medicine, University of Illinois Chicago, College of Medicine at Peoria, Peoria, USA; 3 Cardiology, University of Illinois Chicago, College of Medicine at Peoria, Peoria, USA; 4 Internal Medicine, University of Illinois College of Medicine at Peoria, Peoria, USA; 5 Cardiology, University of Illinois College of Medicine, Order of St. Francis Medical Centre, Peoria, USA

**Keywords:** cost effectiveness analysis, primary percutaneous coronary intervention (pci), coronary artery disease, symptoms, symptoms severity scale

## Abstract

Introduction

Atherosclerotic coronary artery disease (CAD) is the major cause of mortality in the USA. CAD requiring percutaneous coronary intervention (PCI) can have a wide spectrum of presentations. We reviewed the cost of admission and PCI at the tertiary care center stratified for different CAD presentation types.

Methods

We performed a retrospective study of 7,389 patients undergoing coronary angiogram at our facility from 2015 to 2017. Patients were selected from CathPCI registry. Chart review was done for readmission and death data. Cost data were provided by the finance department. Patients going for coronary artery bypass surgery (CABG) were excluded. We split the patients based on their need for PCI. Cost analysis was based on CAD presentation types (No symptoms, atypical symptoms, stable angina, unstable angina, NSTEMI [non-ST segment elevation myocardial infarction], STEMI [ST-segment elevation myocardial infarction]). Adjusted linear regression was run for the outcomes. Primary outcomes were 30-day readmission and death. The secondary outcome was cost of admission.

Results

The final sample size was 6,403. The mean age was 65.6 years (SD: 12.5; male: 63.8%). 2444 required PCI (38%; p < 0.001). PCI group had lower mean age (62.5 years; SD: 12.3, p<0.001) with lower BMI (30.6 vs 31.1, p=0.015). PCI group had significantly lower odds for 30-day readmission (OR: 0.63; CI: 0.45-0.89; p=0.009) and 30-day mortality (OR:0.60; CI: 0.41-0.89; p = 0.011). A severe presentation increased the odds of getting PCI. Cost of admission was higher in all groups receiving PCI.

Conclusions

PCI group had better 30-day readmission and mortality rates. PCI increases the cost of admission in all CAD types.

## Introduction

Atherosclerotic coronary artery disease (CAD) is major cause of death in United States [1]. Percutaneous coronary artery intervention (PCI) remains the most common treatment modality among the treatments of coronary artery disease. More than 600,000 PCIs are performed in the United States each year, the cost associated with these procedures is estimated at around $12 billion [2]. Although the mortality associated with the procedure is low, the readmission rate after the procedure is indicative of the quality of care and determines the cost of care [3]. The main determinants of cost-effectiveness are age and class of angina. PCI is most successful in the case of left ventricular disease and in younger patients [4]. 

For patients with stable angina COURAGE trial compared medical therapy with PCI and found the cost to be higher for the intervention arm [5]. However, a subsequent analysis done for stable angina patients has emphasized that the acceptable cost threshold for improvement in symptoms is difficult to achieve [6]. In patients with unstable angina and non-ST elevation myocardial infarction (NSTEMI) studies comparing the cost-effectiveness of early intervention versus conservative management revealed increased cost associated with the intervention [7]. In STEMI patients the cost-effectiveness of the PCI is reported in terms of length of stay (LOS), 30-day readmission, and mortality associated with the procedures, and these outcomes depend on the location of the lesion. Studies have shown that early discharge (3 days) after PCI is associated with decreased 30-day readmission rate and cost, especially in non-anterior wall myocardial infarction [8].

Based on varying outcomes in different CAD presentation types, it is difficult to calibrate the factors affecting the cost of admission. In this study, we looked at the admission cost associated with different types of coronary artery disease presentations with and without PCI.

The abstract from this article has been published in the National Meeting of Cardiology. SCAI 2019. https://onlinelibrary.wiley.com/doi/10.1002/ccd.28864.

## Materials and methods

Patient population and study design

We performed a retrospective analysis of 7,389 patients who had undergone coronary angiogram at our facility, between January 2015 and December 2017. Institutional Review Board approval was obtained from the office of Human Research at the University of Illinois Chicago at Peoria, IL. Initial patient number was 7,389. We excluded patients with incomplete records, those with missing labs and patients who required CABG. The final sample size was 6,403. We used retrospective data from chart review, and every patient had an electrocardiogram (ECG) available before the procedure. Clinical variables evaluated are listed in Table-1. We split the patients in two categories based on whether the patient received PCI or not. Additionally, we divided patients based on their CAD presentation type at the time of the coronary angiogram. The CAD presentation classes included No symptoms, Atypical symptoms, Stable angina, Unstable angina, NSTEMI, and STEMI.

Outcomes

Primary outcomes were 30-day readmission and mortality. The secondary outcome was cost of admission.

Statistical analysis

Continuous data were reported as mean ± standard deviation (SD) and categorical data as proportions. T-test was utilized to compare continuous variables and the Chi-square test was used for categorical variables. Adjusted statistical analyses were conducted to compare clinical variables. 30-day readmission, 30-day mortality, and admission cost required a multivariate analysis. We used Stata software, v12 for statistical analysis and p-value of less than 0.05 was marked as significant.

## Results

Sample demographics

Of the total 6,403 patients, 2444 (38%) patients received PCI compared with 3959 (62%) who did not receive PCI (No-PCI group) (Table 1). Patients in the PCI group were comparatively younger (mean age of 64.5 vs. 66; p < 0.001) and had lower BMI (30.6 vs. 31.1; p = 0.015). There was a significantly higher proportion of male patients in the PCI group, compared with the No-PCI group (70.9% vs. 59%; p < 0.01).

**Table 1:** Sample demographics. Chi-square test for categorical variables and t-test for continuous variables. # of patients (portion % by column) CAD: coronary artery disease; MI: myocardial Infarction; HF: heart failure; PCI: percutaneous coronary intervention; CABG: coronary artery bypass graft; CVD: cardiovascular disease; PAD: peripheral arterial disease; CCS: Canadian Cardiovascular Society; Non-STEMI: non-ST elevation myocardial infarction.

Continuous variables	All (n = 6403)	PCI (n = 2444)	No PCI (n = 3959)	p-value
Age	65.5 (12.5)	64.5 (12.3)	66.0 (12.5)	<0.001
BMI	30.9 (7.0)	30.6 (6.4)	31.1 (7.4)	0.015
Categorical variables	All (n = 6403)	PCI (n = 2444)	No PCI (n = 3959)	p-value
Male	4083 (63.8)	1732 (70.9)	2351 (59.4)	<0.001
Smoker	1710 (26.7)	796 (32.6)	914 (23.1)	<0.001
Hypertension	4932 (77.0)	1832 (74.9)	3100 (78.3)	0.002
Family history of CAD	657 (10.3)	288 (11.8)	369 (9.3)	0.002
Prior MI	1274 (19.9)	551 (22.5)	723 (18.3)	<0.001
Prior HF	1193 (18.6)	253 (10.3)	940 (23.7)	<0.001
Valve surgery	127 (1.9)	41 (1.7)	86 (2.2)	0.168
Prior PCI	1492 (23.3)	703 (28.7)	789 (19.9)	<0.001
Prior CABG	641 (10.0)	200 (8.2)	441 (11.1)	<0.001
Current dialysis	186 (2.9)	53 (2.2)	133 (3.4)	0.006
Prior CVD	926 (14.5)	334 (13.7)	592 (14.9)	0.155
Prior PAD	840 (13.1)	301 (12.3)	539 (13.6)	0.135
Chronic lung disease	1158 (18.1)	389 (15.9)	769 (19.4)	<0.001
Diabetes	2165 (33.8)	785 (32.1)	1380 (34.8)	0.024
Prior cardio shock	99 (1.5)	65 (2.7)	34 (0.8)	<0.001
Prior cardiac arrest	192 (3.0)	111 (4.5)	81 (2.0)	<0.001
CAD presentation				<0.001
1: No symptoms	740 (11.6)	44 (1.8)	696 (17.6)	
2: Atypical Symptoms	725 (11.3)	41 (1.7)	684 (17.3)	
3: Stable angina	1377 (21.5)	317 (12.9)	1060 (26.8)	
4: Unstable angina	1285 (20.1)	538 (22.0)	747 (18.9)	
5: Non-STEMI	1410 (22.0)	775 (31.7)	635 (16.0)	
6: STEMI	866 (13.5)	729 (29.8)	137 (3.5)	

Clinical variables and PCI treatment

**Figure 1:**
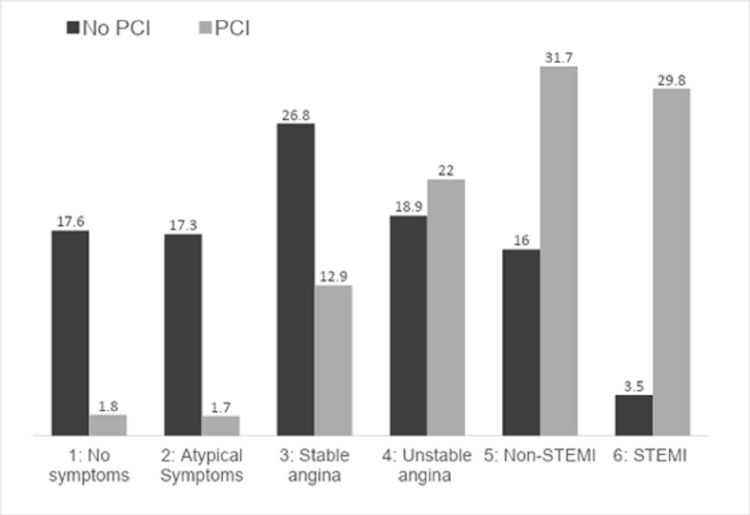
Percutaneous coronary intervention comparison in different coronary artery disease presentation types. Percentage bar graph comparing PCI vs No-PCI ground among CAD presentation classifications.

Patients in the PCI group demonstrated a larger proportion of smokers (32.6% vs 23.1%; p < 0.001) and those with history of CAD (11.8% vs 9.3%; p = 0.002) (Table 1). A significantly larger number of patients in the PCI group reported a history of previous MI (22.5% vs 18.3%; p < 0.001), prior PCI (28.7% vs 19.9%; p < 0.001) and prior cardiogenic shock (2.7% vs 0.8%; p < 0.001). Interestingly, significant number of patients in the No-PCI group had history of hypertension (78.3% vs 74.9%; p = 0.002), chronic lung disease (19.4% vs 15.9%; p < 0.001), diabetes (34.8% vs 32.1%; p = 0.024), and heart failure (23.7% vs 10.3%; p < 0.001). Furthermore, patients in the No-PCI group reported a history of prior CABG at a greater rate (11.1% vs 8.2%; p < 0.001) and were on dialysis more frequently (3.4% vs 2.2%; p = 0.006). The No-PCI group had higher number of patients who presented with no symptoms (17.6% vs 1.8%), atypical symptoms (17.3% vs 1.7%), and stable angina (26.8% vs 12.9%) (Figure 1). The PCI group had a significantly higher number of patients with NSTEMI (31.7% vs 16%), and STEMI (29.8% vs 3.5%) compared with the No-PCI group.

Predictors of PCI treatment

On linear regression analysis (Table 2), male patients had higher odds of receiving PCI compared with female patients (OR: 1.82, CI: 1.6-2.08, p < 0.001). Patients with prior PCI (OR: 1.8, CI: 1.52-2.12, p < 0.001) and prior cardiogenic shock (OR: 1.82, CI: 1.03-3.22, p = 0.040) also had increased odds of receiving PCI. Patients with heart failure (OR: 0.62, CI: 0.52-0.75, p < 0.001), prior CABG (OR: 0.66, CI: 0.53-0.82, p < 0.001), and chronic lung disease (OR: 0.8, CI: 0.68-0.94, p = 0.008) demonstrated decreased odds of receiving PCI.

**Table 2:** Linear regression analysis for clinical variables in patients that receive PCI. CAD: coronary artery disease; MI: myocardial Infarction; HF: heart failure; PCI: percutaneous coronary intervention; CABG: coronary artery bypass graft; CVD: cardiovascular disease; PAD: peripheral arterial disease; CCS: Canadian Cardiovascular Society; Non-STEMI: non-ST elevation myocardial infarction.

Covariates	Odds ratio	p-value	95% CI	
Age	1.00	0.104	1.00	1.01
BMI	1.00	0.779	0.99	1.01
Male	1.82	<0.001	1.60	2.08
Smoker	1.11	0.194	0.95	1.29
Hypertension	1.04	0.630	0.89	1.22
Family history of CAD	1.16	0.135	0.95	1.41
Prior MI	0.94	0.483	0.78	1.12
Prior HF	0.62	<0.001	0.52	0.75
Valve surgery	0.94	0.784	0.59	1.50
Prior PCI	1.80	<0.001	1.52	2.12
Prior CABG	0.66	<0.001	0.53	0.82
Current dialysis	0.74	0.141	0.50	1.10
Prior CVD	1.09	0.335	0.91	1.31
Prior PAD	1.04	0.722	0.86	1.25
Chronic lung disease	0.80	0.008	0.68	0.94
Diabetes	1.00	0.946	0.87	1.14
Prior cardiogenic shock	1.82	0.040	1.03	3.22
Prior cardiac arrest	1.14	0.545	0.75	1.74
CAD presentation (Ref: no symptoms)				
2: Atypical symptoms	0.74	0.207	0.46	1.18
3: Stable angina	1.99	0.002	1.27	3.11
4: Unstable angina	3.70	<0.001	2.37	5.77
5: Non-STEMI	7.31	<0.001	4.72	11.33
6: STEMI	27.98	<0.001	17.41	44.97

**Figure 2:**
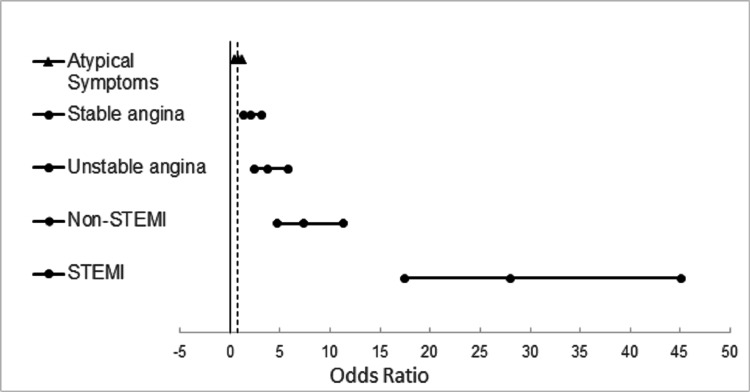
Odds ratio for PCI treatment among CAD presentation type. Non-STEMI: non-ST elevation myocardial infarction; CAD: coronary artery disease; PCI: percutaneous coronary intervention.

Comparison of CAD presentation and PCI rate demonstrated increasing odds of PCI with increased severity of CAD presentation (Figure 2). For example, odds of receiving a PCI were 0.74 in patients with no symptoms at presentation, compared with 27.9 in patients presenting with STEMI (Table 2).

PCI treatment vs 30-day readmission and mortality

Patients in the PCI group showed reduced odds of 30-day readmission (OR: 0.63, CI: 0.45-0.89, p = 0.009) and 30-day mortality (OR: 0.6, CI: 0.41-0.89, p = 0.011) (Table 3). Interestingly, in patients who presented with NSTEMI and received PCI, 30-day mortality did not change (OR: 0.56, CI: 0.30-1.03, p = 0.063).

**Table 3:** Primary outcomes for PCI group as reference. Non-STEMI: non-ST elevation myocardial infarction; CAD: coronary artery disease; PCI: percutaneous coronary intervention.

30-day readmission	Odds ratio	95% CI		p-value
All	0.63	0.45	0.89	0.009
CAD presentation				
1: No symptoms	0.93	0.11	7.89	0.947
2: Atypical Symptoms	NA			
3: Stable angina	1.61	0.29	8.81	0.583
4: Unstable angina	0.92	0.36	2.35	0.857
5: Non-STEMI	0.50	0.31	0.81	0.005
6: STEMI	0.67	0.33	1.37	0.275
30-day mortality	Odds ratio	95% CI		p-value
All	0.60	0.41	0.89	0.011
CAD presentation				
1: No symptoms	0.35	0.07	1.68	0.192
2: Atypical Symptoms	NA			
3: Stable angina	NA			
4: Unstable angina	1.22	0.26	5.75	0.803
5: Non-STEMI	0.56	0.30	1.03	0.063
6: STEMI	0.60	0.31	1.16	0.127

PCI treatment vs cost

**Figure 3:**
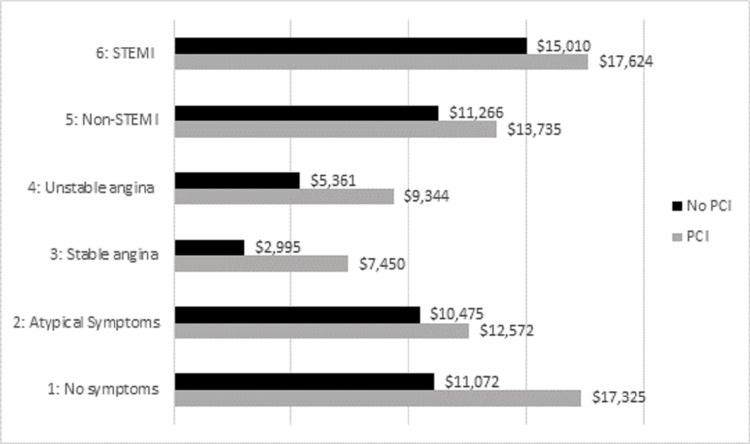
Cost associated with PCI vs No-PCI among CAD presentation types. Non-STEMI: non-ST elevation myocardial infarction; CAD: coronary artery disease; PCI: percutaneous coronary intervention.

PCI treatment added on average $4,731 to the total cost of admission ($12,753 vs $8,022; p < 0.001) (Table 4). Notably, PCI treatment among patients that presented with stable angina ($7450 vs $2995; p < 0.001), unstable angina ($9,344 vs $5361; p < 0.001), NSTEMI ($13,735 vs $11,266; p < 0.001), and STEMI ($17,624 vs $15,010; p = 0.025) were all associated with increased cost (Figure 3).

**Table 4:** Generalized linear model with log link and gamma distribution analysis of PCI and cost. CAD: coronary artery disease; Non-STEMI: non-ST elevation myocardial infarction; PCI: percutaneous coronary intervention.

Cost	PCI	No PCI	Difference	95% CI		p-value
All	$12,753	$8,022	$4,731	$3,699	$5,763	<0.001
CAD presentation						
1: No symptoms	$17,325	$11,072	$6,253	-$4,954	$17,459	0.274
2: Atypical Symptoms	$12,572	$10,475	$2,097	-$6,249	$10,444	0.622
3: Stable angina	$7,450	$2,995	$4,455	$3,316	$5,594	<0.001
4: Unstable angina	$9,344	$5,361	$3,983	$2,378	$5,588	<0.001
5: Non-STEMI	$13,735	$11,266	$2,468	$1,300	$3,637	<0.001
6: STEMI	$17,624	$15,010	$2,613	$330	$4,897	0.025

## Discussion

CAD has a spectrum of presentations and mortality risk depends on which end of the spectrum the patient belongs. Regardless, all attempts are made to make sure the diagnosis of coronary artery disease is not missed, particularly with atypical presentations. This leads to a whole gamut of workup which may end up with percutaneous coronary intervention. CAD does have significant social and economic implications.

The overall cost of hospitalization depends, predominantly, whether the patient receives any kind of intervention. Many other factors further contribute to the cost including geographical location, availability of an intervention, which tercile the hospital belongs to, proficiency in complex interventions, etc. Various cost-effective analyses have been done comparing medical management and interventions. A systemic review done by Gholami et al. showed that percutaneous coronary interventions had good results in terms of quality-of-life measures and intermediate initial costs, however, required a higher need for further revascularizations [9]. On the other hand, medical therapy was associated with lower initial costs with lower quality of life measures. Caruba et al. included fifteen randomized control trials and did a network metanalysis and concluded that significant savings in the healthcare expenditure can be achieved by using medical therapy in stable angina patients [10].

So far there has been no study discussing the cost of hospitalization with varied presentations of CAD. Based on our analysis, we had 22.9% of the population with no or atypical symptoms who underwent a coronary angiogram, and only 3.5% received an intervention. The odds for intervention increased consistently with the rest of the presentations, stable angina through STEMI. This was reflected with respect to the cost in our economic analysis. There was no significant difference in the cost for the patients without symptoms or with atypical symptoms whether they received the intervention or not. However, with typical symptoms, there was a definite increase in the cost of the group receiving an intervention. Part of the reason for the increase in cost in the intervention group includes the duration of the stay in the hospital, which is increased as those patients required monitoring post-intervention.

Limitations

Our study is a retrospective analysis with its inherent limitations. Although the percentage of PCI goes up with the more intense presentation of CAD, we had about 1.8% patients in the “No symptoms” group who received PCI; these are likely the patients with occult CAD, presenting for diagnostic coronary angiogram, however because of lack of complete data on the indication, we cannot make this assumption with certainty. In addition, the difference in cost of care was highest for patients with no symptoms ($6253), when they received a PCI; we have limited data to explain the reason for this difference as well. 

30-day readmission was significantly high in the NSTEMI group, we do not have any explanation for this finding. In addition, we did not have enough data points for readmission in patients with atypical symptoms.

## Conclusions

The primary outcome of mortality and 30-day readmission rates were better in the PCI group compared with the No-PCI group. The addition of PCI to coronary angiogram was associated with an increased cost of admission. Notably, PCI treatment among patients that presented with stable angina, unstable angina, NSTEMI, and STEMI was associated with significantly higher cost.
